# Ultrastructural localization and distribution of Nardilysin in mammalian male germ cells

**DOI:** 10.1186/s12610-016-0032-9

**Published:** 2016-04-05

**Authors:** D. Segretain, J. Gilleron, J. N. Bacro, M. Di Marco, D. Carette, G. Pointis

**Affiliations:** UMR S 1147 Université Paris Descartes, 45 rue des Saint-Pères, 75006 Paris, France; Université de Versailles Saint-Quentin-en-Yvelines (UVSQ), Versailles, 78000 France; INSERM U 1065, Université Nice Sophia-Antipolis, 151 route Saint-Antoine de Ginestière BP 2 3194, 06204, Nice, cedex 3 France; Institut de Mathématiques et de Modélisation de Montpellier (I3M), UMR CNRS 5149 Université Montpellier, CC 51; 4 place Eugène Bataillon 34095, Montpellier, cedex 5 France

**Keywords:** Nardilysin, Gold immunohistochemistry, Spermiogenesis, Flagellum, Nardilysine, Immunohistochimie couplée à l’or colloïdal, Spermiogénèse, Flagelle

## Abstract

**Background:**

NRD convertase, also termed Nardilysin, is a Zn^++^ metalloendopeptidase that specifically cleaves the N-terminus of arginine and lysine residues into dibasic moieties. Although this enzyme was found located within the testis, its function in male reproduction is largely unknown. In addition, the precise distribution of this enzyme within germ cells remains to be determined.

**Methods:**

To answer these questions, we developed an immuno-gold electron microscopy analysis to detect Nardilysin at ultrastructural level in mice. In addition, we performed a quantitative analysis of these gold particles to statistically estimate the distribution of Nardilysin in the different subcellular compartments of differentiating late spermatids/spermatozoa.

**Results:**

Expression of Nardilysin in wild-type mice was restricted to germ cells and markedly increased during the last steps of spermiogenesis. In elongated spermatids, we found the enzyme mainly localized in the cytoplasm, more precisely associated with two microtubular structures, the manchette and the axoneme. No labelling was detected over the membranous organelles of the spermatids. To test whether this localization is dependent of the functional microtubules organization of the flagella, we analysed the localization into a specific mouse mutant *ebo/ebo (ébouriffé)* known to be sterile due to an impairment of the final organization of the flagellum. In the *ebo/ebo*, the enzyme was still localized over the microtubules of the axoneme and over the isolated cytoplasmic microtubules doublets. Quantification of gold particles in wild-type and mutant flagella revealed the specific association of the enzyme within the microtubular area of the axoneme.

**Conclusions:**

The strong and specific accumulation of Nardilysin in the manchette and axoneme suggests that the enzyme probably contributes either to the establishment of these specific microtubular structures and/or to their functional properties.

## Résumé

**Objectifs:**

La NRD convertase aussi appelée Nardilysine, une Zn^++^ metalloendopeptidase qui clive spécifiquement dans la région N terminale des résidus arginine et lysine des sites dibasiques, est impliquée dans la transformation/maturation des proprotéines. Le but de cette étude est de localiser cette enzyme durant la spermiogénèse afin de comprendre son rôle au cours de la maturation des spermatides.

**Méthodes:**

La Nardilysine est révélée par immunohistochimie au niveau ultrastructural chez des souris contrôles fertiles et chez un mutant stérile (ébouriffé : *ebo/ebo*). Des analyses quantitatives sont effectuées par comptage des grains d’or colloïdal qui permettent de détecter la localisation spécifique de l’enzyme au cours de la croissance des spermatides dans des régions particulières.

**Résultats:**

L’expression de la Nardilysine chez les souris sauvages et stériles *ebo/ebo* est limitée aux cellules germinales avec une augmentation significative dans les étapes ultimes de la spermiogénèse. L’enzyme est fortement exprimée dans le cytoplasme des spermatides allongées et dans les structures microtubulaires, la manchette et le flagelle. Aucun marquage n’est observé au niveau des organites cellulaires des spermatides. Chez le mutant *ebo/ebo,* dont le flagelle est anormal, l’enzyme est toujours présente sur les doublets de microtubules du flagelle. La quantification des particules d’or chez la souris sauvage et chez le mutant révèle une association spécifique de l’enzyme avec les microtubules du flagelle.

**Conclusions:**

L’accumulation spécifique de la Nardilysine au niveau de la manchette et du flagelle suggère que cette enzyme pourrait contribuer à l’établissement de ces structures microtubulaires particulières et/ou à leurs propriétés fonctionnelles.

## Background

N-arginine dibasic convertase (NRD) also termed Nardilysin [1], a Zn^++^ metalloendopeptidase which shows cleavage specificity for the N-terminus of arginine residues in dibasic sites of various peptide substrates [2–4], was proposed to participate in the proprotein processing [5, 6].

Nardilysin is expressed in many tissues and recent studies suggest that it can be involved in several human physiological and pathological situations. Indeed it plays an essential role in the homeostasis of body temperature by tuning brown adipose tissue production through a regulation of UCP1 expression [7]. It was also associated with axonal maturation in the central nervous system (CNS) [8] and could be implicated in human pathologies such as Alzheimer disease, Down syndrome, schizophrenia, mood disorders, alcohol abuse, heroin addiction [7–10]. Other studies reported its potential role as a marker of type1 diabetes progression [11]. Nardilysin was also found to be involved in oesophageal cancer cell invasion and in gastric cancer via the induction of inflammatory actors [12, 13].

Abundant in many endocrine tissues, Nardilysin was found in the testis [14–16], where high levels have been detected. Spermatogenesis is a complex process, which produces highly differentiated cells, the spermatozoa. During spermiogenesis, young round spermatids elongate, reorganize their organelles as also their chromatin [17–19] to become the highly sophisticated elongated spermatids, the future spermatozoa. The cytoskeleton is implicated in such changes. In addition to the normal array of microtubules found in most somatic cells, the spermatids contain specific microtubular structures such as the manchette, a microtubular organelle essential for sperm head shaping, and the flagellum/axoneme organization [20–22]. Moreover, numerous enzymatic activities, such as protein kinase C (PKC) and the MAST 205 kinase, were found to play a role in germ cells protein maturation [23–30] that is essential for sperm maturation [31, 32]. From these data, it has been evidenced that a complex array of enzymatic elements is implicated in the remodelling of the elongated spermatids. The possibility that Nardilysin actively participates in this process has been suggested by our previous observations that this enzyme is specifically expressed in germ cells during late stages of spermatogenesis and mostly associated with microtubular structures [33].

The purpose of the present study was to determine with more precision at the ultrastructural/electron microscopic level, the exact location of the Nardilysin within maturing spermatids and its evolution during spermiogenesis in relation to the morphological events which take place during this morphological process. Using a specific mouse mutant, *ebo/ebo*, characterized by a defect in flagellar organization leading to an arrest in spermatozoa maturation [34], we confirmed that the fine ultrastructural distribution of Nardilysin is independent of flagellar microtubules organization. The results indicate that Nardilysin was distributed throughout the spermatid cytoplasm, preferentially associated with microtubules main components of manchette and axoneme.

## Methods

### Animals and experimental procedures

All animal studies were conducted in accordance with French legislation and guidelines for proper use of animals in research. Testis and epididymis from four adult C57/BL6 mice and from four *ebo/ebo mice* mutants [34] were used in this study. Immunohistochemistry analyses were performed at electron microscopic level.

### Ultrastructural electron microscopic (EM) analysis

For EM studies, ten tissue pieces of the testis and epididymis (cauda and caput) per animal were obtained from intracardiac fixative perfused animals. They were fixed with 4 % paraformaldehyde in Phosphate Buffer Saline (PBS, 0.1 M; pH 7.4) containing 0.1 % glutaraldehyde. The free aldehydes were quenched with ammonium chloride (NH_4_Cl- 50 mM) as previously described [35]. After an en block staining with Uranyl acetate (5 % in PBS) for 1 h at room temperature, three randomly selected small tissue pieces per animal were dehydrated in cold alcohol and embedded in Lowicryl K4M. Thin sections were deposited on nickel grids coated with Formvar and processed for ultrastructural gold-immunohistochemistry (100 sections per tissue pieces). Briefly, grids were first floated on PBS containing normal goat serum 3 % (NGS, from British Biocell International), followed by incubation with the primary antibody. The NRD convertase specific rabbit polyclonal antiserum [2] was used at 1/750 final dilution, diluted in PBS plus 1 % NGS for 2 h at room temperature. After several rinses in PBS, grids were deposited on the second antibody (goat anti-rabbit coupled to 15 nm gold particles (GAR-15 from TEBU). Finally, the tissue sections were briefly counterstained with Uranyl acetate and Lead citrate. Thirty randomly-selected sections per tissue pieces were examined at 60 kV with a Phillips EM-CM 10 at the Institut Alfred Feyssard (CNRS, avenue de la Terrasse, Gif sur Yvette 91190, France).

To determine the amount of background, we generated control sections that followed exactly the same protocol described above, except that the primary antibody was omitted. In control sections, only few gold particles were found in the cytoplasm and in the nucleus of the cells (5.3 ± 0.82) and in the lumen of the tubules (4.7 ± 0.66). No gold particle was observed in the flagellum. Because the number of gold particles detected in the NRD-labelled sections (with the primary antibody directed against-NRD) was quite similar in the nucleus of the cells (4.5 ± 0.58) and in the lumen of the tubules (3.9 ± 0.52) as compared to control sections, we assumed that this staining correspond to unspecific signal. On the contrary, since we found a high number of gold particles on the flagellum of NRD-stained sections (17.1 ± 1.2) compared to no gold in the control condition, we considered this signal as specific. The low number of gold particles present over Sertoli, myoid and Leydig cells corresponded to the background level.

### Morphometrical analysis

The quantification of the gold particles per surface unit (expressed in pixel) was realized on ninety EM testis and epididymis sections per animal (X.11500). Gold particles were counted in spermatids at various steps of spermiogenesis. In addition, we counted the number of gold particles in the various levels of the spermatozoa flagellum (mid-piece, principal and distal portions). Because, in each tissue section, a mix of various orientations of the flagellum, such as longitudinal, oblique or cross sections were represented, only cross sections of the flagellum were selected for analysis. The quantitation of the results was performed with the Visilog 4.15 image analysis system (Noesis SA; les Ulis, 91140, France). A specific miniprogram was developed for this part of the work (courtesy of B. Prilleux).

### Statistical analysis

The aim of the work is to analyze the distribution of gold particles in defined regions of the flagellum (R1 to R6). Clearly, the statistical analysis is related to global group’s mean comparison and multiple group mean comparisons. Standard methods such as variance analysis are not adequate because of data heteroscedasticity. Our statistical approach was thus based on nonparametric statistics by using median values rather than means. Comparisons between two groups were made by the Mann–Whitney test, while for global comparison between three or more groups, the Kruskal-Wallis test was used. In both tests, the level of significance is equal to 5 %.

Following a Kruskal-Wallis test that rejects the null hypothesis, one may conduct tests of equality of all pairs of rank sums in order to ascertain which are different from the others. One-at-a-time individual comparisons for pairs of rank sums may be used for this purpose. However, such an approach would not be adjusted for multiple inference; the error rate controlled is the Type I error rate for each individual test. When pairwise comparisons are made, multiple comparison procedures attempt to control the probability of making at least one Type 1 error. These procedures protect the experimenter against declaring too many significant tests, that means insure that the probability of making at least one Type I error is controlled.

Briefly, in non parametric minimal signification difference approach, when the global comparison’s test (Kruskal-Wallis test) is significant, we consider that the medians from the groups *i* and *j* differ at a signification level α if the absolute value of the mean ranks of these two groups are greater than a value which depends (among others factors) on the numbers of observations in each group, the ranks sums of each group when mixed and a value related to α (via the Student probability law). This procedure is rightful if the global Kruskal-Wallis test is significant and the pairwise comparisons are made at a level that is not smaller than that of the global test.

More precisely, in agreement with [36], the decision rule at level is the following: the groups i and j are considered to be significantly different if

$$ \left|{s}_t^{\hbox{'}}\kern1em -\kern1em {s}_j^{\hbox{'}}\right|\kern1.5em >\kern1.5em {t}_{N-q\kern0.5em ,a}\kern1em \sqrt{\frac{\left({S}_r^2-C\right)\left(N-1-T\right)\left({n}_i+{n}_j\right)}{n_i{n}_j\left(N-q\right)\left(N-1\right)}} $$

where *q* is the number of groups which are globally compared, *N* is the total number of observations while *n*_*i*_ and *n*_*j*_ are respectively the numbers of observations in each group *i* and *j*,

${s^{\hbox{'}}}_i=\frac{s_i}{n_i}.$ and ${s^{\hbox{'}}}_j=\frac{s_i}{n_i}.$ where *s*_*i*_ and *s*_*j*_ designate respectively the ranks sums for the groups *i* and *j,*

*t*_*N* − *q* ,*a*_ the value such that a Student test would be significant at 100α% level,

others quantities are directly related to the Kruskal-Wallis test: $C=\frac{1}{4}N{\left(N+1\right)}^2,$

$T=\left(N-1\right)\frac{S_q^2-C}{S_r^2-C}$ with ${S}_q^2={\displaystyle \sum_i\left(\frac{S_i^2}{n_i}\right)}$ and *S*_*r*_^2^ designates the total squared ranks sum.

## Results

Stages of spermatogenesis and steps of spermiogenesis were classified in the mouse as described by Oakberg (1956) [37].

### Adult wild-type mouse

**Fig. 1:**
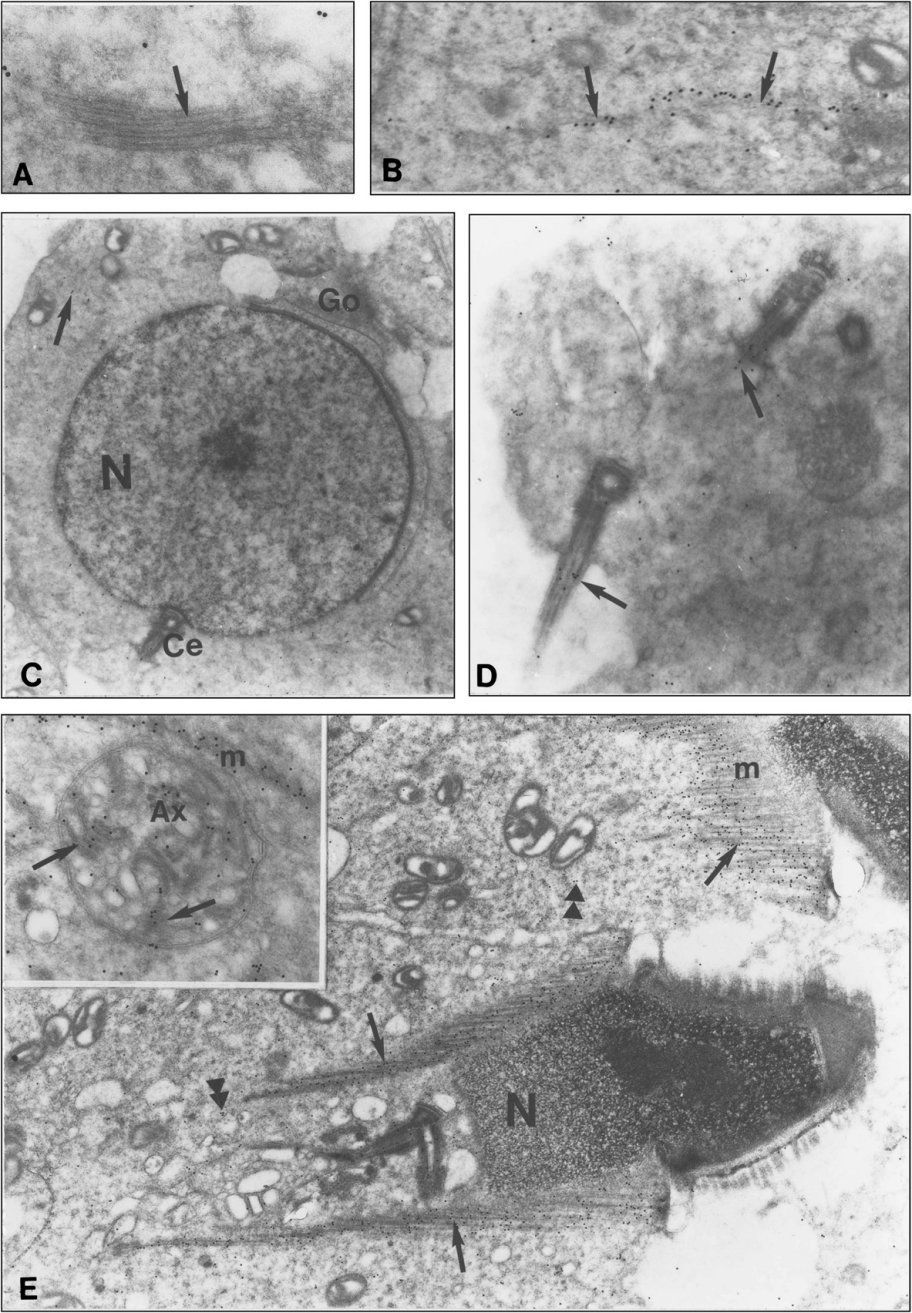
Immunogold electron micrographs of Nardilysin in wild-type mouse in round and elongated spermatids. **a** In pachytene spermatocytes the cluster of microtubule is unlabelled (X 61 000). **b** In round spermatid at step 2–3 cytoplasmic microtubules are decorated by gold particles (X 60 000). **c** Step7 spermatid showing typical nucleus (N), acrosomic granule associated with the Golgi apparatus (G) and at the opposite face the pair of centrioles (Ce). The cytoplasm contains few gold particles (X 12 000). **d** High magnification of an abnormal step 6–7 spermatid which contains cytoplasmic labelling as also some particles over the growing flagellum (arrow) (X 25 000). **e** Step 13 spermatid revealing the elongated nucleus (N) and the caudally located cytoplasm surrounding the flagellum. If the cytoplasm contains some NRD convertase particles, the microtubule manchette (m) is strongly labelled (arrows), as also the distal centriole (arrow). No labelling was present over the mitochondria (X 14 000). In the Inset, at the same step, the gold particles are distributed over the axoneme, the periaxonemal vesicles and the anlagen of the fibrous sheath (arrowhead) (X 31 000)

In the testis, at the ultrastructural level, the immunoreactive signal for Nardilysin, identified by the presence of gold particles, was localized exclusively to the germ cell layer. No labelling was observed in spermatogonia and spermatocytes, nor in dividing germ cells, at the level of the mitotic spindle. Signal was also absent on the specific clusters of microtubules in spermatocytes at stage VII of spermatogenesis (Fig. 1a). Control immunogold-labelled sections were in all cases devoid of gold particles.

At the first steps of mouse spermiogenesis (steps 1 to 7) during the Golgi and Cap phase, the immunoreactivity appeared over the cytoplasm of the spermatids (Fig. 1b, c, d). At higher magnification, the immunoreactive dots were cytoplasmic and appeared as rows of gold particles underlining small bundle of cytoplasmic microtubules (Fig. 1b). The only noticeable labelling was present over the nascent flagellum, which originates from an elongation of the distal centriole, out of the cell cytoplasm with the accompanying cell plasma membrane. If a low immunoreactivity was present in round spermatids cytoplasm, the growing flagellum presented more gold particles as compared to the cytoplasm (Fig. 1c, d). Later, at steps 8 to 12 of spermiogenesis, during the elongation phase, the immunolabelling was present over the caudal cytoplasm (Fig. 1e). If the gold particles were mostly located over the entire spermatid cytoplasm, with a highest reactivity as compared to round cells (compare Fig. 1c with Fig. 1e), a marked signal appeared over the microtubules of the manchette (Fig. 1e). In face view, the sheath-like structure with a curtain appearance contained numerous gold particles located over its microtubular component (Fig. 1e and inset). A similar strong immunoreactivity was depicted on the growing flagellum. At this level, only the elongating portion of the distal centriole exhibited numerous gold particles. Sometimes, the proximal centrioles, as well as the centriolar adjuncts, were slightly immunopositive (Fig. 1c, d and e). However, the membranous components of the spermatids, such as endoplasmic reticulum (ER) cisternae, radial body and annulate lamellae as also the membranous invaginations of the Sertoli cell processes remained always unlabelled (Fig. 1e). At these elongating steps, around the growing flagellum isolated by plasma membrane invaginations, numerous membranous components took place (Fig. 1e, inset). The periaxonemal vesicles and the electron-dense components present in the periaxonemal cytoplasm, which look like as anlagens of Fibrous Sheath (FS), were always labelled (Fig. 1e, inset).

**Fig. 2:**
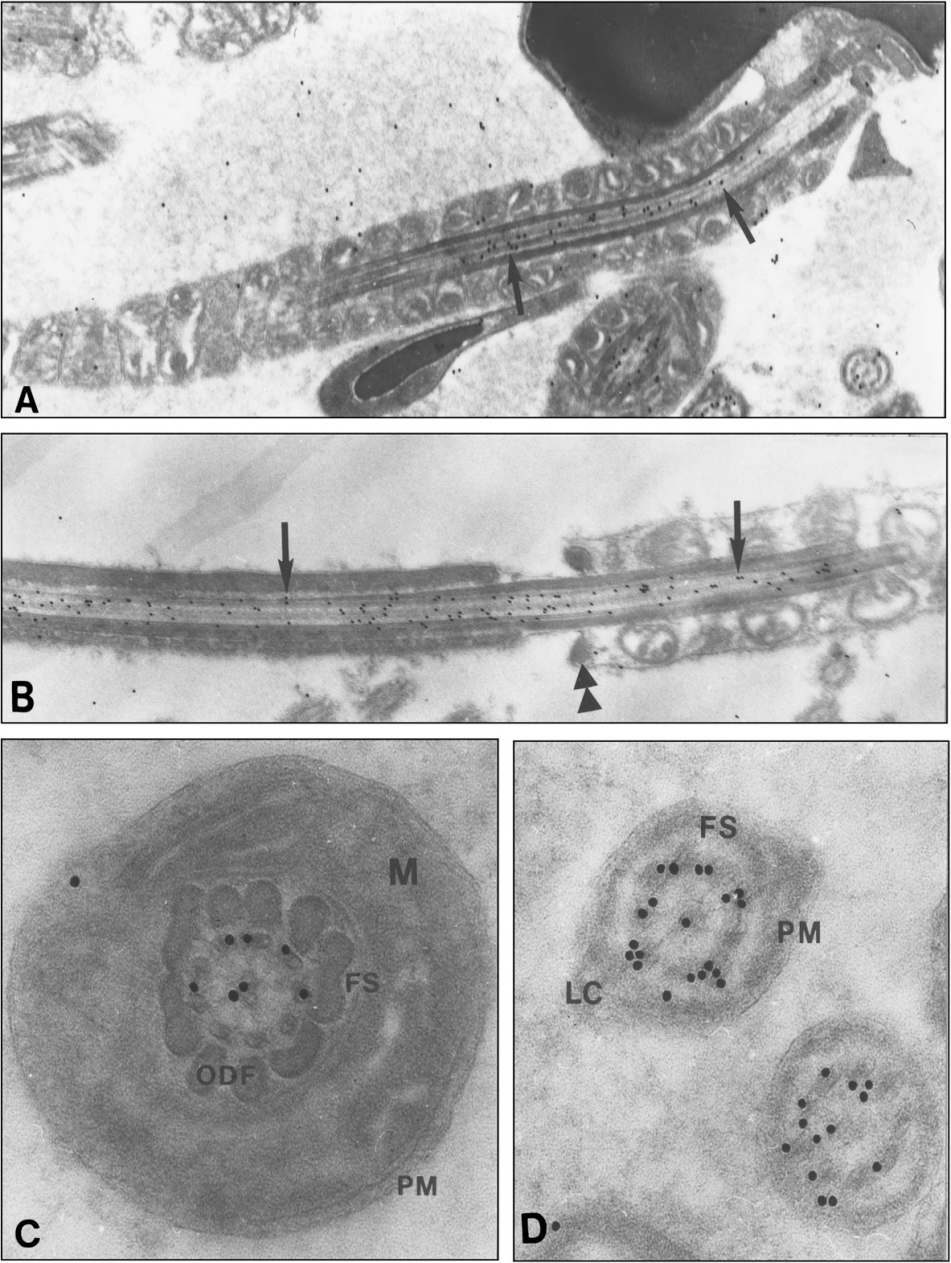
Immunogold electron micrographs of Nardilysin in wild-type mouse in late spermatids. **a** and **b** Longitudinal sections of step 16 spermatid. **a** Mid piece region of the flagellum, close to the Head. Gold particles are mostly distributed over the central microtubular component (arrow) (X 31 000). **b** At the beginning of the proximal region, the strong labelling of the microtubules is obvious (arrows). No labelling was noticed on the annulus (X 31 000). In the caput epididymis, cross sections of the flagellum, present in the lumen of the tubules, are immunoreactive. **c** In the midpiece region, the gold particles are distributed over the microtubules doublets and the central singulets. PM, plasma membrane; ODF, outer dense fibers; FS, fibrous sheath; M, mitochondria (X 85 500). **d** In the distal portion of the flagellum, the intense labelling of the microtubules underlines their specific organization (X 84 000)

Later, during the maturation phases, at steps 13 to 15, the labelling of the cytoplasm was still barely visible, as was the strong immunoreactivity of the flagellum (Fig. 1e, inset). Finally, at step 16, during sperm release, while the head and the neck of the spermatozoa were unlabelled, the three main regions of the tail and the cytoplasmic region of the residual bodies were highly immunoreactive (Fig. 2a, b). Examination of longitudinal sections of the flagellum showed that the centrioles, inserted in the implantation fossa, were poorly labelled (Fig. 2a). The gold particles appeared distributed over the microtubules and rarely over the other components of the mid-piece region (Fig. 2a). Similarly, the labelling was present on the microtubules which formed the central portion of the proximal and distal end of the tail (Fig. 2b). Interestingly, a helicoidal distribution of the gold particles could be depicted from time to time on the longitudinal sections. This specific distribution of the labelling was tested with a computer analysis, specifically developed for the flagellum (courtesy of H. Delacroix, CNRS, Gif sur Yvette). The precise organization observed did not correspond to date to a specific arrangement over some components like radial spokes, dynein arms or nexin links. At the level of the junction between the mid-piece and the proximal portion of the tail, the annulus was unlabelled (Fig. 2b).

In cross sections of the epididymis, gold particles were mostly located over the microtubular components of the flagellum (Fig. 2c). However, few particles were present over the outer dense fibers (ODF) and the mitochondria. In appropriate sections, the gold particles were observed over the microtubules doublets and singlets (Fig. 2c). Then, they mimicked the regular pattern of organization of the tubular components. In the distal region of the tail spermatozoa, a strong labelling was obvious (Fig. 2d). At the same time, the immunoreactivity of the Residual Bodies was mostly located over the cytoplasm of these degenerating portions of late spermatids (data not shown).

**Fig. 3:**
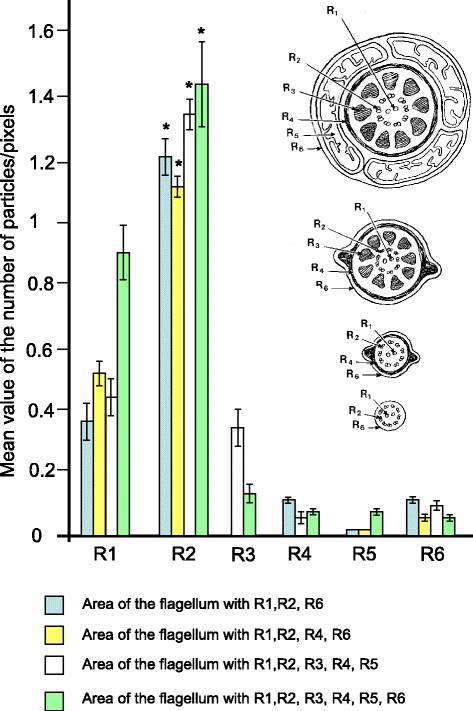
Quantification of Nardilysin staining in the late spermatids at the testis level, step 16. Number of particles per defined area (R1, R2, R3, R4, R5, R6 expressed in pixels). A drawing represent the various cross section of the flagellum associated with the above region (R) considered. Counting of gold particles per defined region reveals their location mostly over microtubules. * Pvalue <0.05

Semi-quantitative analysis of gold particles per surface area (expressed in pixels square) confirmed the strong labelling of the elongated spermatids as compared to round spermatids (Fig. 3). The highest distribution of Nardilysin was mainly observed when the microtubule manchette was formed (i.e. during the elongation process). The counting of the gold particles per surface area, within the various predefined regions of the growing flagellum, revealed that the region R2, which possesses doublets of microtubules, presented the highest distribution of the enzyme as compared to any other regions. In addition, in comparison with the four main regions of the flagellum (i.e. mid-piece, mid or central and proximal and distal end), morphometrical analysis showed that the main labelled portions of the flagellum are R1 and R2, which contain the microtubules (Fig. 3). Statistical analysis confirmed that the microtubule-containing areas were more densely labelled compared to other predefined areas (P < 0.01).

### Adult male mouse mutant (*ebo/ebo*)

**Fig. 4:**
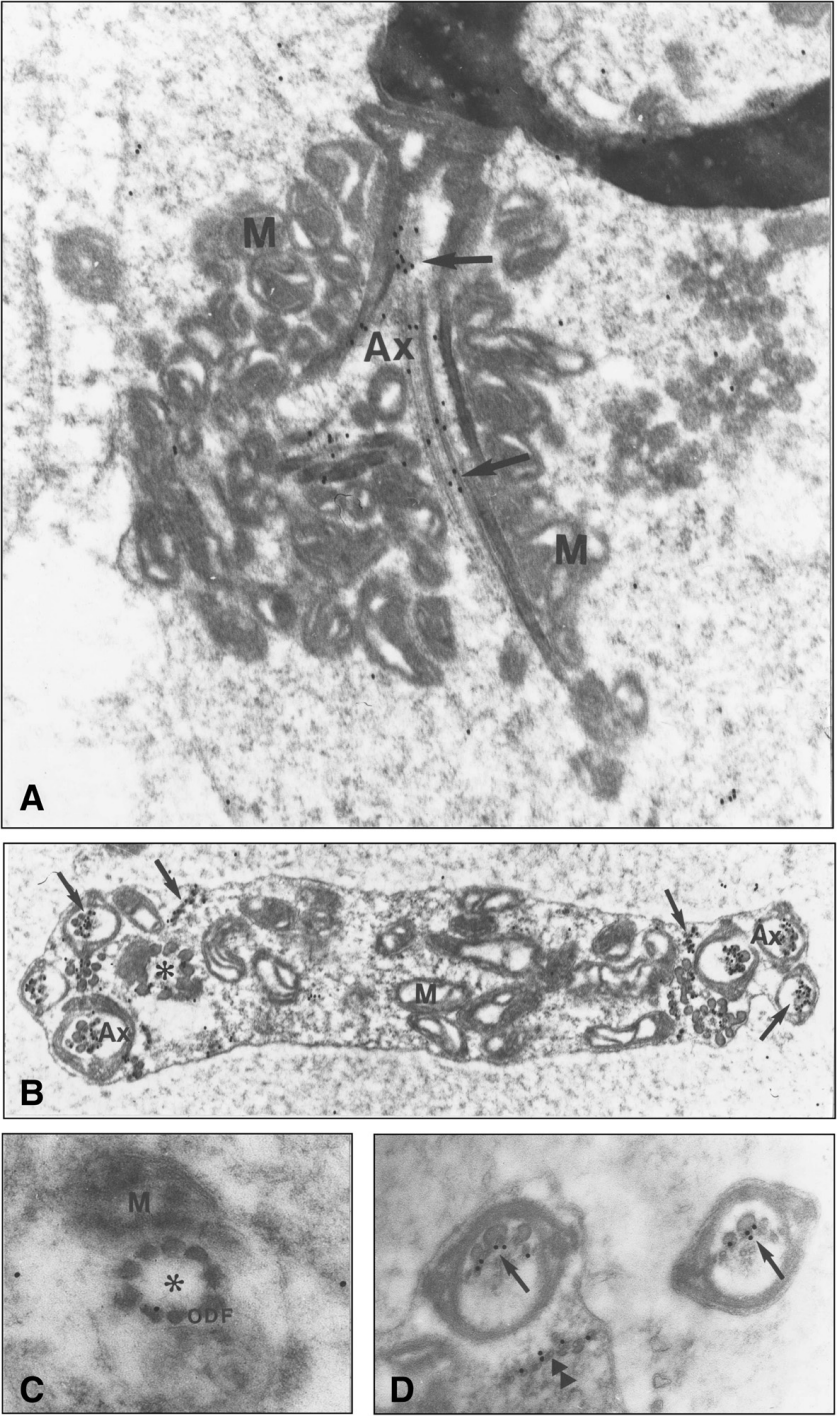
Immunogold electron micrographs of Nardilysin in the caput epididymis of mutant mouse (*ebo/ebo*) spermatozoa. **a** Longitudinal section of an abnormal midpiece showing the misorganization of the mitochondria (M) and the disrupted axoneme (Ax). Gold particles are present on the microtubules of the axoneme (arrows) (X 26 500). **b** Cross section of a flagellar loop, demonstrating numerous cross section of the axoneme in a common cytoplasm. The gold particles underline the microtubules doublets in and out of the axoneme (arrows). Mitochondria (M) (X 26 500). **c** High magnification of a cross section of the flagellum of picture B (noted by asterisk). The ODF are well noticed, whereas the microtubules and the immunolabelling are absent (X 85 500). **d** In cross section of the flagella, the gold particles are only present over the microtubules, inside (arrow) or outside (double arrowhead). (X 63 000)

The use of the *ebo/ebo* mutant mouse, where the major defect occurs at the level of the flagellum formation [34], allowed studying the precise ultrastructural distribution of Nardilysin. As previously described in this mutation, the major defect observed in mouse mutant *ebo/ebo* mostly concerned the organization of the mitochondria of the mid-piece region, concomitant with abnormalities of the microtubules of the flagellum (Fig. 4a, b, d). In longitudinal sections of spermatozoa of the *ebo/ebo* mutants, the misorganization of the mitochondria of the mid-piece was quite obvious (Fig. 4a). The gold particles were solely located over the microtubules of the flagellum as described in the wild-type mouse.

**Fig. 5:**
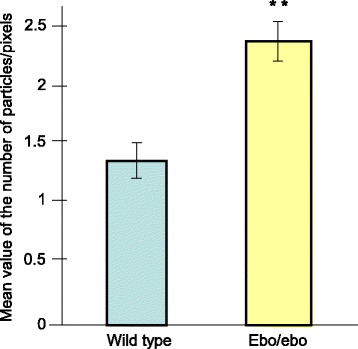
Quantitative analysis of the distribution of the gold particles associated with Nardilysin in wild-type and *ebo/ebo* mutant. ** Pvalue <0.02

In cross sections of several loops of the flagellum in a common cytoplasm, where part of the microtubules doublets were missing and/or disorganized, the gold particles were located over the tail portion containing microtubules doublets (Fig. 4b). Moreover, in the cytoplasm of this atypical flagellum, some immunoreactive signals were present over the isolated microtubule doublets interspersed throughout the cytoplasm. In appropriate sections, some flagella, free of microtubules, were then mostly/solely delineated by the typical organization of the ODF (Fig. 4c). In such sections, no labelling was observed, reinforcing the observation that Nardilysin was only present over the microtubules and/or surrounding it. The most characteristic features were the presence of gold particles over a row of microtubules doublets, out of the flagellum. Such isolated doublets were delineated by the labelling (Fig. 4d). The counting of the gold particles per surface area on the various portions of the cross sections of the *ebo/ebo* flagellum in the epididymis, compared with the wild-type spermatozoa in the same region, revealed that in any cases, the labelling was restricted to the microtubules doublets and showed a significant increase (P < 0.01) in the mutant mice (Fig. 5).

## Discussion

There is now compelling evidence that NRD or Nardilysin, a Zn^++^ metalloendopeptidase, is highly expressed in the testis [4, 33]. However, its precise location and the physiological role of this enzyme during spermiogenesis have not been to date reported. By developing immuno-electron microscopy approaches associated with statistical analysis, we clearly demonstrate here that Nardilysin is primarily associated with spermatids microtubules. This is also in accordance with an accessory investigation showing that in a specific mouse mutant *ebo/ebo* with spermatogenesis arrest due to impairment of the final organization of the flagellum [34], Nardilysin was still associated with microtubule structures.

Previously, by using immunohistochemistry and western blot analysis approaches, some of us reported that Nardilysin is mainly located in the spermatid layer of the seminiferous epithelium [33]. The current data demonstrate for the first time both the precise location of Nardilysin, primarily associated with spermatid microtubules and the timing of the enzyme expression during spermiogenesis. Electron microscopy allowed us to demonstrate that a specific signal for Nardilysin was detected early in the growing flagellum of the round spermatids suggesting the existence of a close relationship between the expression of the enzyme and the microtubule doublets and singlets composing the growing axoneme. This possibility is also supported by the examination of the mutant *ebo/ebo*, which displays abnormalities mostly at the level of the flagellum [34].

Many proteins have been located at the spermatid levels [26, 29, 38–40] but only few of them are present at the level of the growing flagellum. In addition, while other enzymes, such as PKC [31, 41], show an irregular distribution along the axoneme, the current data reveal that Nardilysin was present all along this structure with sometimes a helicoidal distribution. Previous immunofluorescence studies reported the existence of an association between dynein and tubulin in the manchette [42] suggesting that interactions between microtubules and various proteinaceous compounds, including enzymes, are obvious. Thus, it is conceivable that Nardilysin can interact not only with microtubules but also with other partners such as dynein arms. At this level and along the flagellum where dynein arms are abundant, Nardilysin could play a role in the formation (synthesis) and/or degeneration or renewing of the dynein arms. This is supported by a recent study demonstrating that Nardilysin could be involved in the structuration of the microtubules network as also with their movements [43]. This possibility is also in agreement with the current observations in the mutant *ebo/ebo*, which shows a strong immunolabeling in isolated microtubules doublets. In addition, as it was previously proposed for the serine/threonine protein kinase MAST 205 [32], it can be hypothesized that Nardilysin may function on specific unknown substrates that regulate the formation, movement or disassembly of the manchette during spermiogenesis.

The present ultrastructural study confirmed our previous study at the light microscopic level [33], that Nardilysin accumulated in the cytosol while no gold particles were detected on any membranous structures such as the Golgi apparatus and ER cisternae. A possible explanation for the lack of labelling on ER and Golgi saccules could be that the antibody obtained against the mature protein recognized only this mature form in the cytosol. Another explanation could be that Nardilysin is synthesized by the cells as a precursor or native form at this level, rendering it undetectable, and then the mature form can be present in the cytoplasm, more or less coupled with microtubules. Previous studies reported that Nardilysin is expressed at a time when transcriptional activity in the haploid germ cell nucleus has terminated [16]. Thus as reported for FS and ODF proteins corresponding to cytoskeletal adjuncts of the flagellum [44, 45], it can be hypothesized that Nardilysin transcripts must first be stored in the spermatid before being translated. By in situ hybridization, it has been observed that NRD convertase transcripts were detected only in elongating germ cells [33] thus confirming the ultimate appearance of the enzyme concomitantly with the mRNAs.

The present study shows that Nardilysin was present on spermatid microtubules but not on spermatocyte microtubules. In addition, Nardilysin was not found associated with microtubules mitotic spindle during cellular division. Altogether, our results suggest that all microtubules are not equivalent in term of proteins and/or enzymes composition. Previous data suggested that the formation of such specific microtubules should be the result of the expression of specific genes and/or a “cascade” of genes and then proteins [46]. Therefore, it is likely that Nardilysin could participate in the maturation of microtubules specifically in spermatid in order to mature the microtubules onto a very specialized structure required for the function of the flagellum. Further analyses will be necessary to sort out whether other components of this macromolecular structure are specific to flagellar microtubules and to demonstrate their importance.

## Conclusion

Nardilysin should play a role in positioning and/or formation of some components in relation with microtubules of the manchette and of the flagellum. The presence of this enzyme only in spermatids leads to suggest that specific enzymatic events must be implicated in spermatids maturation. The use of a mutant allows confirming such proposal for Nardilysin expression. It could be relevant to determine the fine role of the enzyme in the complex structure and movement of the microtubules associated with theirs partners. Several questions arise from the current investigation: i) Does Nardilysin play a key role in the final positioning of microtubules, in control and in non-physiological situations? This could be examined by developing new imaging techniques such as superresolution microscopy to ascertain their relationship with other microtubules environment. ii) Does the detection of Nardilysin only on spermatid microtubules point the fact that these cytoskeletal compounds, as their microenvironment, are different from those of the other cells types of the seminiferous epithelium?

## Abbreviations

CNS: central nervous system
ER: endoplasmic reticulum
FS: fibrous sheath
GAR: Goat anti-rabbit coupled to gold particles
MAST205 kinase: 205-kDa manchette microtubule-associated serine/threonine protein kinase
MMP2: Matrix metalloproteinases
NRD: N-arginine dibasic convertase
ODF: outer dense fiber
PBS: phosphate buffer saline
PKC: protein kinase C
Zn: zinc

## References

[1] Hospital V, Prat A (2004). Nardilysin, a basic residues specific metallopeptidase that mediates cell migration and proliferation. Protein Pept Lett..

[2] Chesneau V, Pierotti AR, Barré N, Créminon C, Tougard C, Cohen P (1994). Isolation and characterization of a dibasic selective metalloendopeptidase from rat testes that cleaves at the amino terminus of arginine residues. J. Biol. Chem..

[3] Cohen P, Pierotti AR, Chesneau V, Foulon T, Prat A, Barrett AJ (1995). N-Arginine dibasic convertase. Methods in Enzymology : Metalloendopeptidase.

[4] Chow KM, Ma Z, Cai J, Pierce WM, Hersh LB (2005). Nardilysin facilitates complex formation between mitochondrial malate dehydrogenase and citrate synthase. Biochim Biophys Acta.

[5] Pierotti AR, Prat A, Chesneau V, Gaudoux F, Leseney AM, Foulon T, Cohen P (1994). N-Arginine dibasic convertase, a metalloendopeptidase as a prototype of a class of processing enzyme. Proc. Nat. Acad. Sci. USA.

[6] Ma Z, Chow KM, Csuhai E, Hersh LB (2004). The use of proteolysis to study the structure of nardilysin. Arch Biochem Biophys..

[7] Hiraoka Y, Matsuoka T, Ohno M, Nakamura K, Saijo S, Matsumura S, Nishi K, Sakamoto J, Chen PM, Inoue K, Fushiki T, Kita T, Kimura T, Nishi E (2014). Critical roles of nardilysin in the maintenance of body temperature homoeostasis. Nat Commun.

[8] Ohno M, Hiraoka Y, Lichtenthaler SF, Nishi K, Saijo S, Matsuoka T, Tomimoto H, Araki W, Takahashi R, Kita T, Kimura T, Nishi E (2014). Nardilysin prevents amyloid plaque formation by enhancing α-secretase activity in an Alzheimer’s disease mouse model. Neurobiol Aging..

[9] Bernstein HG, Steiner J, Bogerts B, Stricker R, Reiser G (2014). Nardilysin, ADAM10, and Alzheimer’s disease: of mice and men. Neurobiol Aging.

[10] Bernstein HG, Stricker R, Dobrowolny H, Steiner J, Bogerts B, Trübner K, Reiser G (2013). Nardilysin in human brain diseases: both friend and foe. Amino Acids..

[11] Uraoka N, Oue N, Sakamoto N, Sentani K, Oo HZ, Naito Y, Noguchi T, Yasui W (2014). NRD1, which encodes nardilysin protein, promotes esophageal cancer cell invasion through induction of MMP2 and MMP3 expression. Cancer Sci..

[12] Radichev IA, Maneva-Radicheva LV, Amatya C, Parker C, Ellefson J, Wasserfall C, Atkinson M, Burn P, Savinov AY (2014). Nardilysin-dependent proteolysis of cell-associated VTCN1 (B7-H4) marks type 1 diabetes development. Diabetes..

[13] Kanda K, Komekado H, Sawabu T, Ishizu S, Nakanishi Y, Nakatsuji M, Akitake-Kawano R, Ohno M, Hiraoka Y, Kawada M, Kawada K, Sakai Y, Matsumoto K, Kunichika M, Kimura T, Seno H, Nishi E, Chiba T (2012). Nardilysin and ADAM proteases promote gastric cancer cell growth by activating intrinsic cytokine signalling via enhanced ectodomain shedding of TNF-α. EMBO Mol Med..

[14] Foulon T, Cadel S, Chesneau V, Draoui M, Prat A, Cohen P (1996). Two novel metallopeptidases with a specificity for basic residues: functional properties, structure and cellular distribution. Ann N Y Acad Sci..

[15] Hospital V, Prat A, Joulie C, Chérif D, Day R, Cohen P (1997). Human and rat testis express two mRNA species encoding variants of NRD convertase, a metalloendopeptidase of the insulinase family. Biochem J..

[16] Winter AG, Pierotti AR (2000). Gene expression of the dibasic-pair cleaving enzyme NRD convertase (N-arginine dibasic convertase) is differentially regulated in the GH3 pituitary and Mat-Lu prostate cell lines. Biochem J..

[17] Tanaka H, Baba T (2005). Gene expression in spermiogenesis. Cell Mol Life Sci..

[18] Rathke C, Baarends WM, Awe S, Renkawitz-Pohl R (1839). Chromatin dynamics during spermiogenesis. Biochim Biophys Acta..

[19] Nair M, Nagamori I, Sun P, Mishra DP, Rhéaume C, Li B, Sassone-Corsi P, Dai X (2008). Nuclear regulator Pygo2 controls spermiogenesis and histone H3 acetylation. Dev Biol..

[20] O’Donnell L, O’Bryan MK (2014). Microtubules and spermatogenesis. Semin Cell Dev Biol..

[21] Kierszenbaum AL, Rivkin E, Tres LL (2007). Molecular biology of sperm head shaping. Soc Reprod Fertil Suppl..

[22] Sperry AO (2012). The dynamic cytoskeleton of the developing male germ cell. Biol Cell..

[23] Wan HT, Mruk DD, Tang EI, Xiao X, Cheng YH, Wong EW, Wong CK, Cheng CY (2014). Role of non-receptor protein tyrosine kinases in spermatid transport during spermatogenesis. Semin Cell Dev Biol.

[24] Gungor-Ordueri NE, Mruk DD, Wan HT, Wong EW, Celik-Ozenci C, Lie PP, Cheng CY (2014). New insights into FAK function and regulation during spermatogenesis. Histol Histopathol..

[25] Ibanez CF, Pelto-Huikko M, Sader O, Ritzen EM, Hersh LB, Hakfelt T, Persson H (1991). Expression of choline acetyltransferase mRNA in spermatogenic cells results in an accumulation of the enzyme in the post-acrosomal region of mature spermatozoa. Proc. Natl. Acad. Sci. USA.

[26] Westhoff D, Kamp G (1997). Glyceraldehyde 3-phosphate dehydrogenase is bound to the fibrous sheath of mammalian spermatozoa. J. Cell. Sci..

[27] Seidah NG, Day R, Hamelin J, Gaspar A, Collard MW, Chretien M (1992). Testicular expression of PC-4 in the rat: molecular diversity of a novel germ cell-specific Kex2/subtilisin-like proprotein convertase. Mol Endoc.

[28] Cadel S, Pierotti AR, Foulon T, Créminon C, Barré N, Segretain D, Cohen P (1995). Aminopeptidase-B in the rat testes : isolation, functional properties and cellular localization in the seminiferous tubules. Mol Cell. Endoc..

[29] Sibony M, Segretain D, Gasc JM (1994). Immunolocalization and in situ hybridization detection of the germinal Angiotensin-Converting Enzyme isoform in murine testes : specific stage expression during spermatogenesis. Biol. Reprod..

[30] Macleod J, Mei X, Erlichman J, Orr GA (1994). Association of the regulatory subunit of a type II cAMP-dependent protein kinase and its binding proteins with the fibrous sheath of rat sperm flagellum. Eur. J. Biochem..

[31] Kalina M, Socher R, Rotem R, Naor Z (1995). Ultrastructural localization of protein kinase C in human sperm. J. Histochem and Cytochem..

[32] Walden PD, Cowan NJ (1993). A novel 205 kilodalton testis-specific serine/threonine protein kinase associated with microtubules of the spermatid manchette. Mol. Cell. Biol..

[33] Chesneau V, Prat A, Segretain D, Hospital V, Dupaix A, Foulon T, Jégou B, Cohen P (1996). NRD-Convertase : a putative processing endoprotease associated with the axoneme and the manchette in late spermatids. J. Cell. Sci..

[34] Lalouette A, Lablack A, Guenet JL, Montagutelli X, Segretain D (1996). Male sterility caused by sperm cell-specific structural abnormalities in Ebouriffé, a new mutation of the house mouse. Biol. Reprod..

[35] Gilleron J, Carette D, Fiorini C, Dompierre J, Macia E, Denizot JP, Segretain D, Pointis G (2011). The large GTPase dynamin2: a new player in connexin 43 gap junction endocytosis, recycling and degradation. Int J Biochem Cell Biol..

[36] Sprent P, Smeeton NC. Applied nonparametric statistical methods. Book series Texts in statistical science. Edition Chapman et Hall/CRC press, 2007, 4th edition. ISBN 9781584887010

[37] Oakberg EF (1956). Duration of spermatogenesis in the mouse and timing of the stages of the cycle of the seminiferous epithelium. Am. J. Anat..

[38] Weinman S, Ores-Carton C, Escaig F, Feinberg J, Puszkin S (1986). Calmodulin immunoelectron microscopy: redistribution during ram spermatogenesis and epididymal maturation. II. J Histochem Cytochem..

[39] Nayernia K, Böhm D, Topaloglu O, Schlüter G, Engel W (2001). Rat transition nuclear protein 2 regulatory region directs haploid expression of reporter gene in male germ cells of transgenic mice. Mol Reprod Dev..

[40] Ferrer M, Cornwall G, Oko R (2013). A population of CRES resides in the outer dense fibers of spermatozoa. Biol Reprod.

[41] Naor Z, Breitbart H (1997). Protein kinase C and mammalian spermatozoa acrosome reaction. Trends Endocrinol Metab..

[42] Yoshida T, Ioshii SO, Imanaka-Yoshida K, Izutsu K (1994). Association of cytoplasmic dynein with manchette microtubules and spermatid nuclear enveloppe during spermiogenesis in rats. J. Cell. Sci.

[43] Tsang WY, Dynlacht BD (2013). CP110 and its network of partners coordinately regulate cilia assembly. Cilia..

[44] Fawcett DW (1975). The mammalian spermatozoon. Dev Biol..

[45] Clermont Y, Oko R, Hermo L (1990). Immunocytochemical localization of proteins utilized in the formation of outer dense fibers and fibrous sheath in rat spermatids: an electron microscope study. Anat Rec..

[46] Dezelee S, Bras F, Contamine D, Lopez-Ferber M, Segretain D, Tenninges D (1989). Molecular analysis of ref(2)P, a drosophila gene implicated in sigma rhabdovirus multiplication and necessary for male fertility. EMBO. J..

